# Supplementary material for: Murine Alveolar Macrophages Rapidly Accumulate Intranasally Administered SARS-CoV-2 Spike Protein leading to Neutrophil Recruitment and Damage
Source: bioRxiv. 2023 Dec 4:2023.03.13.532446. Originally published 2023 Mar 14. Preprint. [Version 2] doi: 10.1101/2023.03.13.532446 (PMC10120727; doi:10.1101/2023.03.13.532446)
Supplement: 1 [file NIHPP2023.03.13.532446V2-supplement-1.pdf]

bioRxiv preprint doi: https://doi.org/10.1101/2023.03.13.532446; this version posted December 4, 2023. The copyright holder for this preprint (which was not certified by peer review) is the author/funder. This article is a US Government work. It is not subject to copyright under 17 USC 105 and is also made available for use under a CC0 license.

Supplementary Material

Figure 1-figure supplement 1. Preparation of SARS-CoV-2 proteins.

Figure 1-figure supplement 2. Confocal micrographs were taken for staining control and to analyze neutrophil fragmentation caused by SARS-CoV-1 Spike VLPs, comparing them to delta Env VLPs.

Figure 2-figure supplement 1. Intranasal SARS-CoV-2 Spike protein administration to K18-hACE2 mice.

Figure 3-figure supplement 1. Quantification of lung permeability and neutrophil recruitment.

Figure 4-figure supplement 1. Quantification of neutrophil count in cremaster muscle.

Figure 4-figure supplement 2. Localization of Spike protein in the liver following intravenous administration.

Figure 4-figure supplement 3. Localization of SARS-CoV-2 Spike protein at other sites following intravenous injection and subcutaneous injection near the inguinal lymph node.

Video 1. Local Spike protein injection results in neutrophil recruitment in the cremaster muscle.

Video 2. Local injection of Spike proteins causes neutrophil fragmentation.

Video 3. Intravenously injected Spike protein outlines liver sinusoids and accumulates on Kupffer cells.

Video 4. Neutrophils undergo NETosis when plated on A549 cells in the presence of Spike protein.

Video 5. hSiglec-5 expressing HEK293 cells bind and endocytosis Spike protein.

bioRxiv preprint doi: https://doi.org/10.1101/2023.03.13.532446; this version posted December 4, 2023. The copyright holder for this preprint (which was not certified by peer review) is the author/funder. This article is a US Government work. It is not subject to copyright under 17 USC 105 and is also made available for use under a CC0 license.

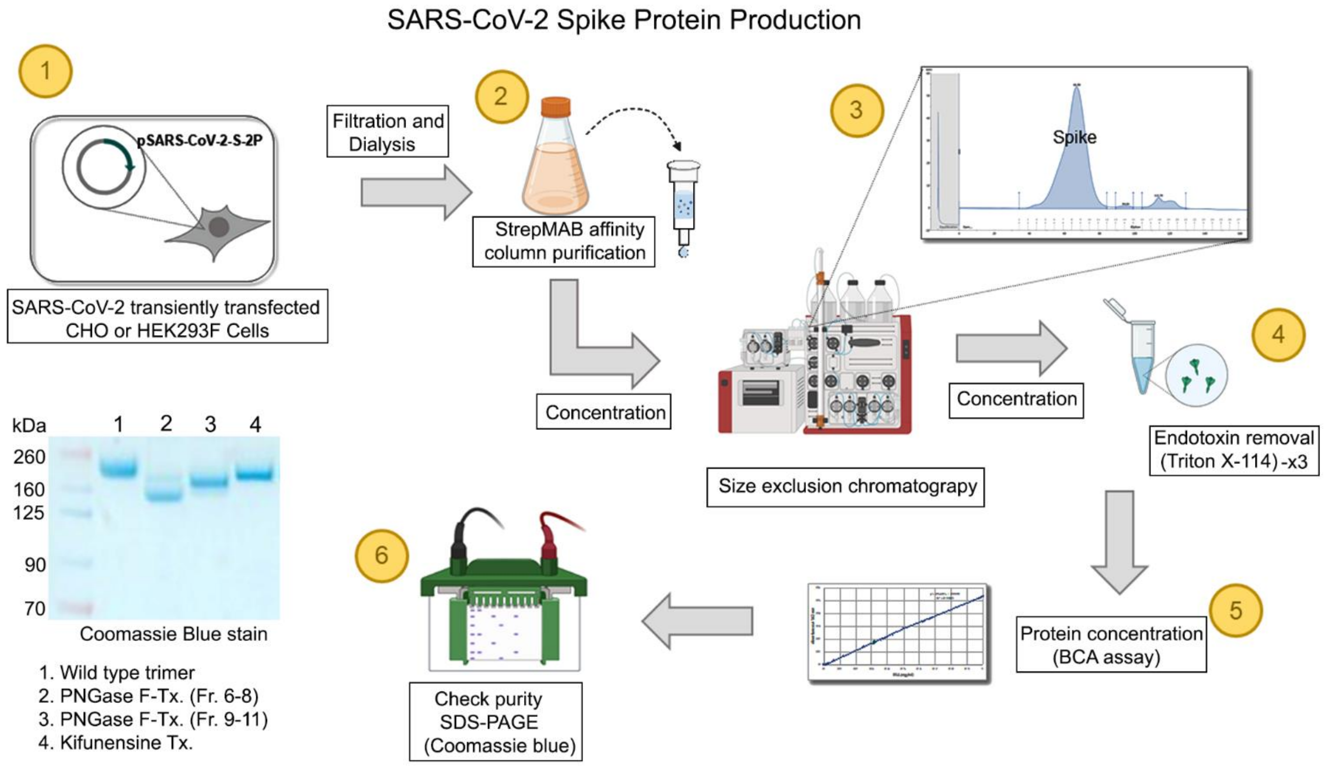

Figure 1-figure supplement 1. Preparation of SARS-CoV-2 proteins. Outline of recombinant proteins production and purification from cultured media of pSARS-CoV-2-S-2P transfected CHO or HEK293F cells. See methods for PNGase F and Kifunensine treatment. Calculated molecular weights: wild type trimer -210 kDa, PNGase F-Tx. (Fr. 6-8)- 146 kDa, PNGase F-Tx (Fx. 9-11)-178 kDa, and Kifunensine Tx.- 197.5 kDa.

bioRxiv preprint doi: https://doi.org/10.1101/2023.03.13.532446; this version posted December 4, 2023. The copyright holder for this preprint (which was not certified by peer review) is the author/funder. This article is a US Government work. It is not subject to copyright under 17 USC 105 and is also made available for use under a CC0 license.

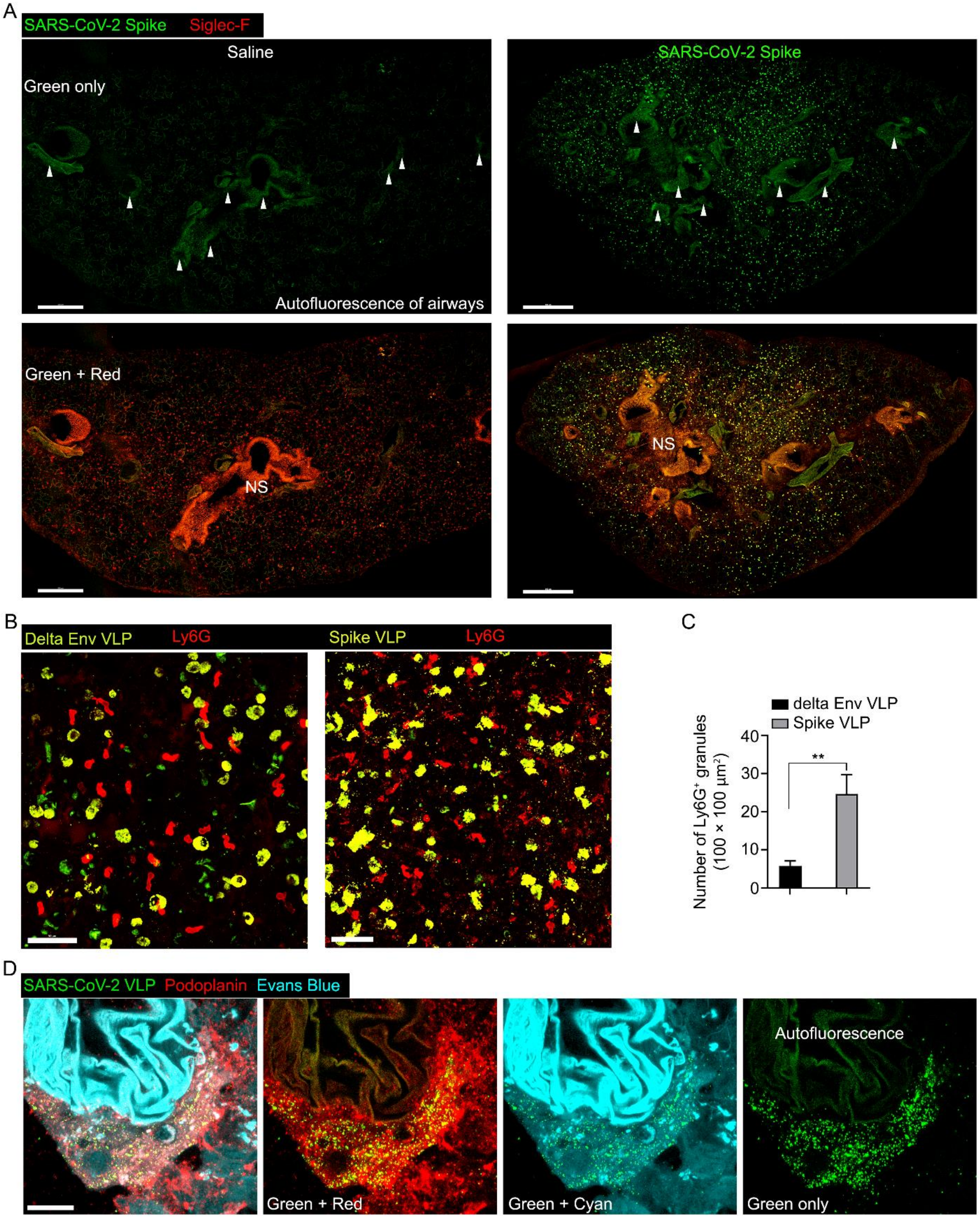

Figure 1-figure supplement 2. Confocal micrographs were taken for staining control and to analyze neutrophil fragmentation caused by SARS-CoV-1 Spike VLPs, comparing them to delta Env VLPs. **(A)** Confocal micrographs compare the negative control (saline) with the positive sample (SARS-CoV-2 Spike, Alexa Fluor 488). Arrowheads in the upper panels indicate autofluorescence background. The nonspecific

bioRxiv preprint doi: https://doi.org/10.1101/2023.03.13.532446; this version posted December 4, 2023. The copyright holder for this preprint (which was not certified by peer review) is the author/funder. This article is a US Government work. It is not subject to copyright under 17 USC 105 and is also made available for use under a CC0 license.

Siglec-F antibody stain background is denoted as 'NS' in the lower panels. Scale bars, 500 µm. **(B)** Confocal micrographs of lungs collected at 3 hours post instillation of SARS-CoV-2 Spike protein (left) or delta envelope (right) VLP (green, GFP) are shown. Infiltrated neutrophils (red, Ly6G) in lung tissue were visualized. Scale bars, 50 µm. (**C**) Neutrophil fragmentation was measured by counting the Ly6G+ granules in five different areas of 100 × 100 μm^2 each. ** $p < 0.01$, paired t-test. (**D)** A confocal micrograph shows a lung lymphatic vasculature visualized with Podoplanin antibody. Fifty microliters of a mixture of Evans blue (cyan) (5 µg) and Spike bearing VLPs (green) (0.5 million counts) were applied to the mouse nose. The first panel presents the merged image of all signals, while the subsequent panels display specific color combinations: green and red in the second, and green and cyan in the third. The fourth panel exclusively exhibits the green signal, with autofluorescence of the lung airway structure indicated. Scale bars, 20 µm.

bioRxiv preprint doi: https://doi.org/10.1101/2023.03.13.532446; this version posted December 4, 2023. The copyright holder for this preprint (which was not certified by peer review) is the author/funder. This article is a US Government work. It is not subject to copyright under 17 USC 105 and is also made available for use under a CC0 license.

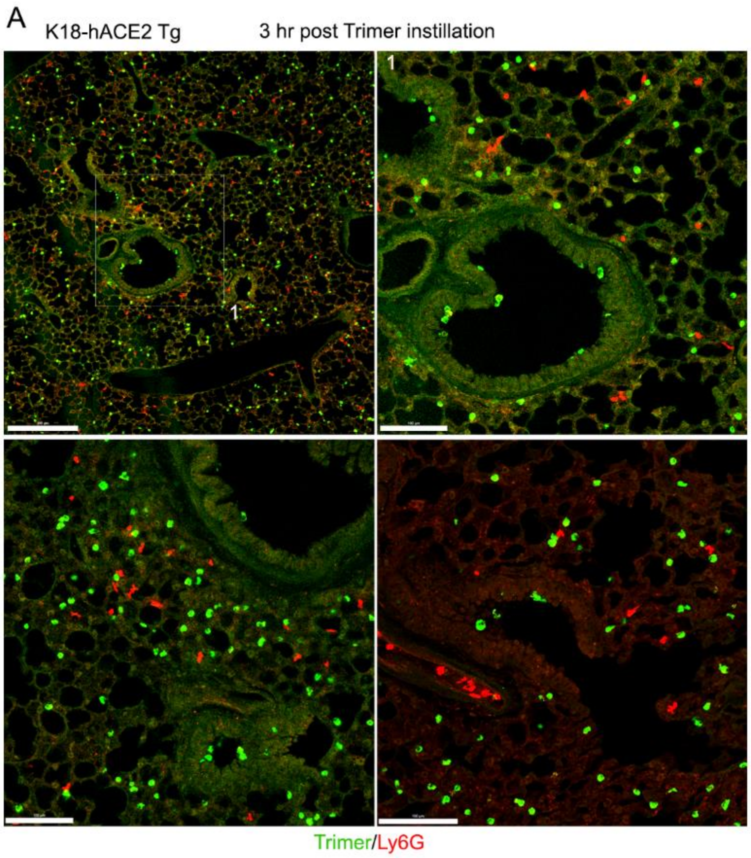

B

	JAX 664	JAX 034860 K18-hACE2
	Spike Trimer (CHO) (n=6)	Spike Trimer (CHO) (n=3)
Neutrophils	3.395 ± 0.916	3.507 ± 0.360
rMono	2.888 ± 0.759	6.830 ± 1.264***
iMono	3.565 ± 0.805	5.257 ± 0.828
AMac	77.467 ± 5.924	87.867 ± 2.609 **
iMac1	9.288 ± 3.234	6.577 ± 0.897
iMac2	13.583 ± 3.234	10.157 ± 0.645
iMac3	31.800 ± 7.223	11.477 ± 1.119 ***
Eosinophils	2.008 ± 0.539	3.803 ± 0.354
CD11b+DC	5.962 ± 1.136	10.497 ± 1.206 ***
CD11b-DC	3.702 ± 0.633	6.667 ± 0.750 **
NK&NKT	1.110 ± 0.176	3.623 ± 0.333 *
B	1.055 ± 0.196	2.403 ± 0.193
T	1.015 ± 0.360	2.710 ± 0.308

Figure 2-figure supplement 1. Intranasal SARS-CoV-2 administration to K18-hACE2 mice. (**A**) Representative confocal images of lung sections 3h post intranasal SARS-CoV-2 Spike protein (3 µg, green). Neutrophils immunostained with Ly6G. (**B**) SARS-CoV-2 Spike protein uptake by lung leukocytes 18 hr following intranasal inoculation. Flow cytometry results from analysis of leukocytes purified from the lungs of WT or K18-hACE2 mice. Data for JAX 664 mice same as shown in figure 2. K18-hACE2 leukocyte values significantly different from those from the JAX 664 mice are indicated, *p < 0.05; **p < 0.01; ***p < 0.001.

bioRxiv preprint doi: https://doi.org/10.1101/2023.03.13.532446; this version posted December 4, 2023. The copyright holder for this preprint (which was not certified by peer review) is the author/funder. This article is a US Government work. It is not subject to copyright under 17 USC 105 and is also made available for use under a CC0 license.

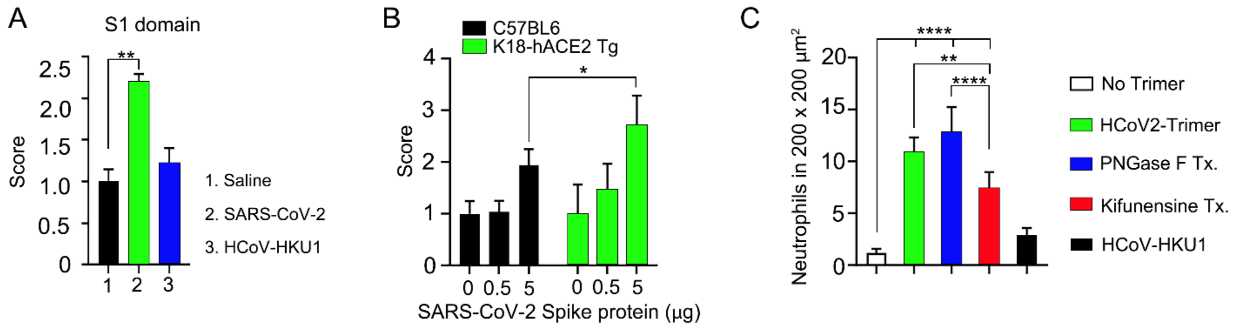

Figure 3-figure supplement 1. Quantification of lung permeability and neutrophil recruitment. **(A)** Evans blue (200 µl of 5 mg/ml in PBS) was intravenously injected 1.5 hours after intranasal Spike administration. The indicated recombinant proteins' S1 domains (3 µg per mouse) were administered. Lungs were collected 1 hour after Evans blue injection. The score was calculated based on the amount of Evans blue dye (in µg/g) in the lungs administered with the Spike protein, divided by the average amount of Evans blue dye in lungs administered with saline. ** $p < 0.01$, One-way ANOVA. **(B)** The permeability of lung vasculature was measured by comparing wild-type mice with K18-hACE2 transgenic mice. The SARS-CoV-2 Spike protein was administered at two different concentrations: 0.5 µg or 5 µg per mouse. * $p < 0.05$, Two-way ANOVA. **(C)** The number of neutrophils was counted in six different areas, each measuring 200 × 200 µm². ****$p < 0.0001$, One-way ANOVA.

bioRxiv preprint doi: https://doi.org/10.1101/2023.03.13.532446; this version posted December 4, 2023. The copyright holder for this preprint (which was not certified by peer review) is the author/funder. This article is a US Government work. It is not subject to copyright under 17 USC 105 and is also made available for use under a CC0 license.

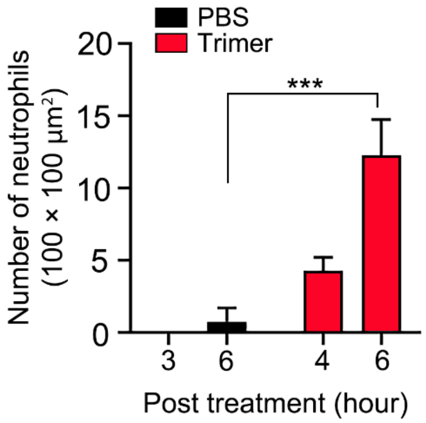

Figure 4-figure supplement 1. Quantification of neutrophil count in cremaster muscle. The number of neutrophils was counted in four 100 × 100 μm² areas. ***$p < 0.001$, Two-way ANOVA.

bioRxiv preprint doi: https://doi.org/10.1101/2023.03.13.532446; this version posted December 4, 2023. The copyright holder for this preprint (which was not certified by peer review) is the author/funder. This article is a US Government work. It is not subject to copyright under 17 USC 105 and is also made available for use under a CC0 license.

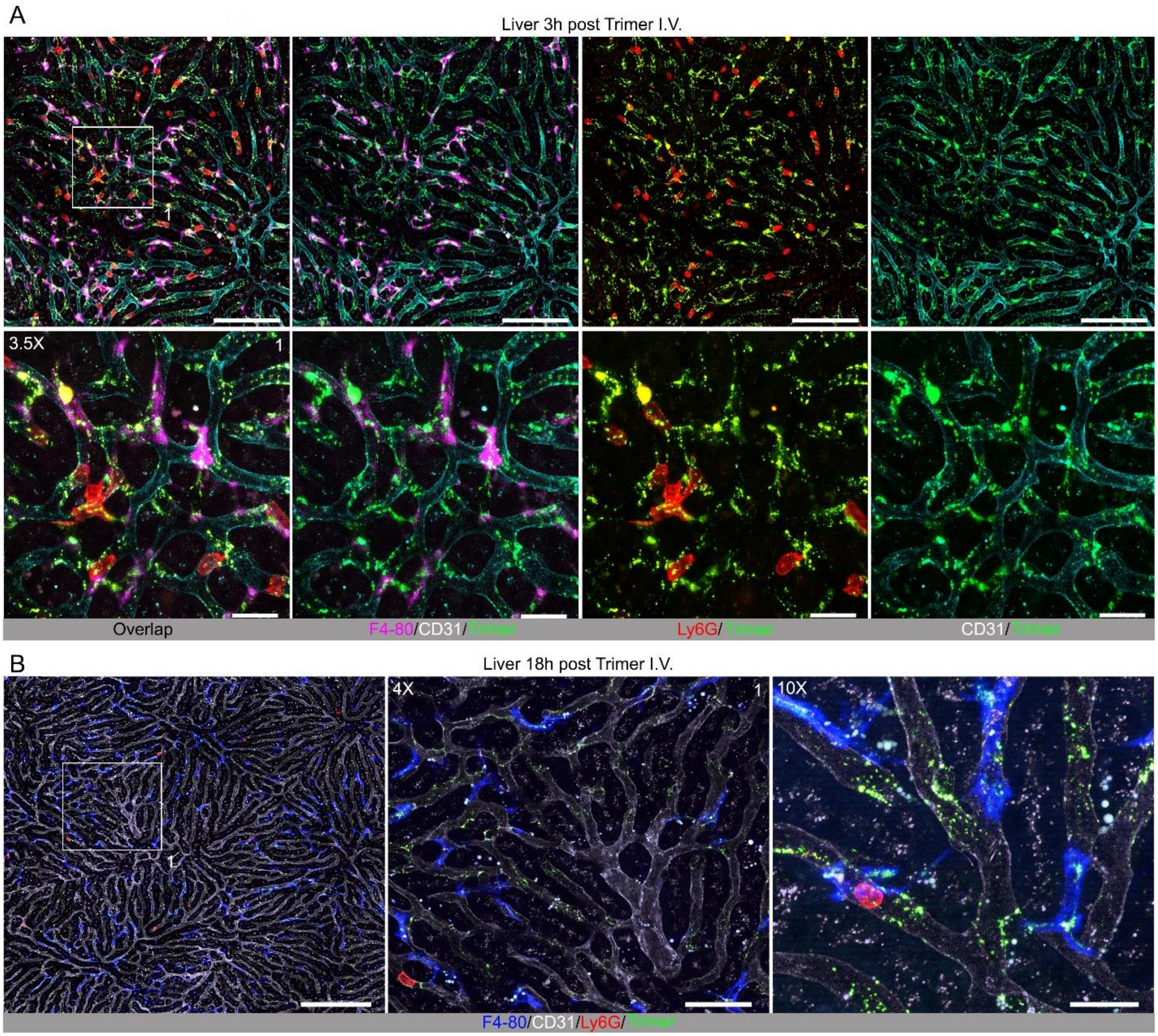

Figure 4-figure supplement 2. Localization of Spike protein in the liver following intravenous administration. **(A)** Confocal micrographs of the liver imaged at 3 hr post intravenous injection of SARS-CoV-2 Spike (Trimer) protein. Spike protein (green), Kupffer cells (magenta, F4/80), and neutrophils (red, Ly6G) shown in liver sinusoid vasculature (cyan, CD31). Antibodies injected 0.3 hr before imaging. ROI-1 (box) (upper left) is enlarged (3.5× magnification) in lower panels. Scale bars, 100 and 20 μm. **(B)** Confocal micrographs of liver imaged at 18 hr post intravenous injection of SARS-CoV-2 Spike protein. Spike protein (green), Kupffer cells (blue, F4/80), and neutrophils (red, Ly6G) are shown in liver sinusoid vasculature (white, CD31). Antibodies injected 0.3 hr before imaging. ROI-1 (box) (left) is enlarged in the middle panel (4× magnification). Enlarged image shows neutrophil contacting Spike protein bearing cells on liver sinusoid endothelium (10× magnification). Scale bars, 200, 50, and 20 μm.

bioRxiv preprint doi: https://doi.org/10.1101/2023.03.13.532446; this version posted December 4, 2023. The copyright holder for this preprint (which was not certified by peer review) is the author/funder. This article is a US Government work. It is not subject to copyright under 17 USC 105 and is also made available for use under a CC0 license.

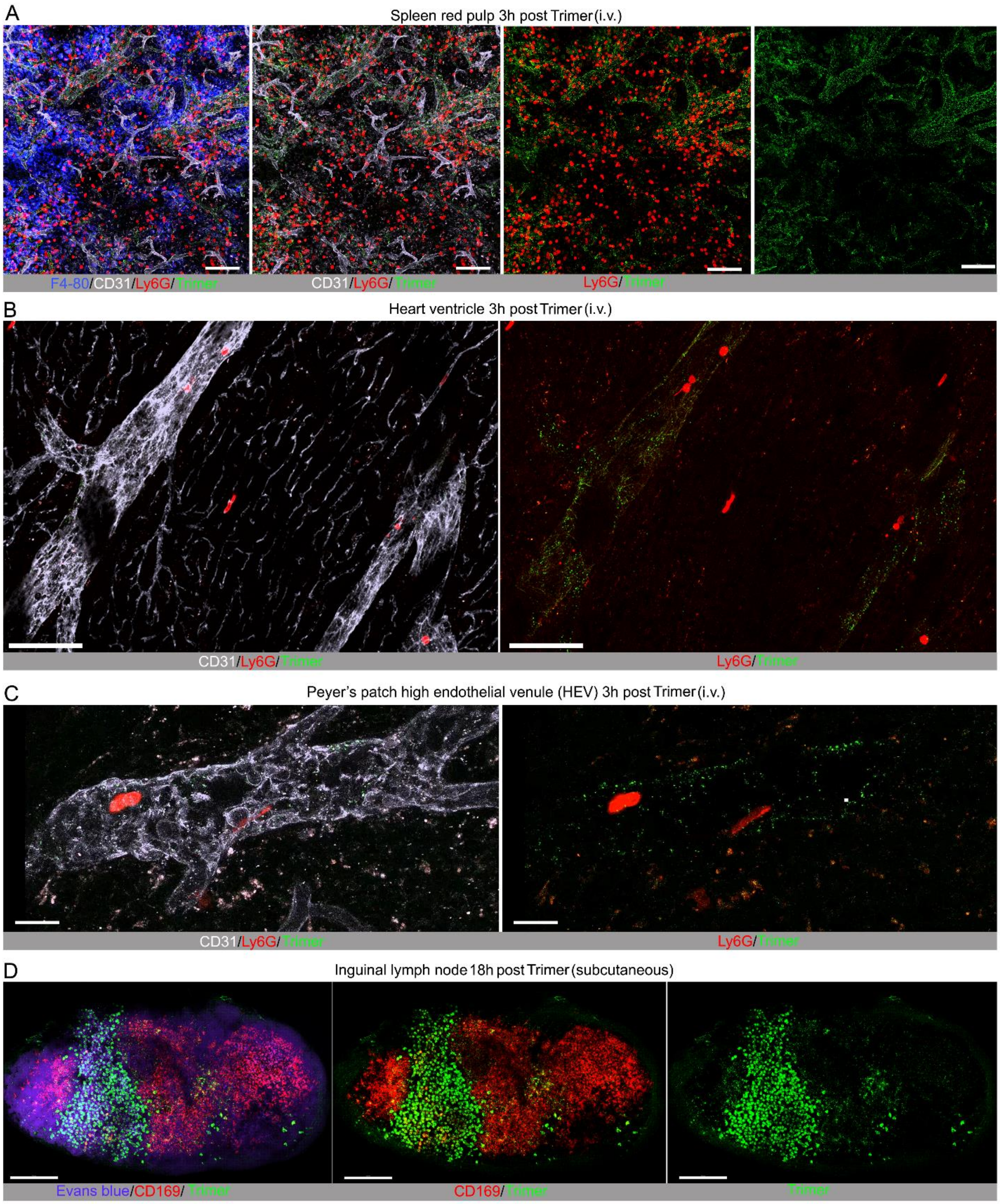

Figure 4-figure supplement 3. Localization of SARS-CoV-2 Spike protein at other sites following intravenous injection and subcutaneous injection near the inguinal lymph node. **(A)** Confocal micrographs of mouse spleen imaged 3 hr post intravenous injection of SARS-CoV-2 Spike (Trimer) protein. Spike

bioRxiv preprint doi: https://doi.org/10.1101/2023.03.13.532446; this version posted December 4, 2023. The copyright holder for this preprint (which was not certified by peer review) is the author/funder. This article is a US Government work. It is not subject to copyright under 17 USC 105 and is also made available for use under a CC0 license.

protein (green), red pulp macrophages (blue, F4/80), and neutrophils (red, Ly6G) visualized in spleen red pulp vasculature (cyan, CD31). Antibodies injected 1 hr before imaging. Scale bars, 100 µm. (**B)** Confocal micrographs of heart ventricle imaged 3 hr post intravenous injection of Spike protein. Spike protein (green) and neutrophils (red, Ly6G) localized in heart blood vessels (white, CD31). Antibodies injected 1 hr before imaging. Scale bars, 100 µm. (**C)** Confocal micrographs of Peyer's patch high endothelial venules (HEV) imaged at 3 hr post iv injection of Spike protein. Spike protein (green) and neutrophils (red, Ly6G) shown in HEV (white, CD31). Antibodies injected 0.5 hr before imaging. Scale bars, 20 µm. (**D)** Confocal micrographs of inguinal lymph node imaged at 18 hr post tail base injection of Spike protein. Spike protein (green) and subscapular sinus macrophages (red, CD169) shown. Subcapsular sinus defined via Evans blue (purple) signal. Antibodies and Evans blue injected 1 hr before imaging. Scale bars, 200 µm.

bioRxiv preprint doi: https://doi.org/10.1101/2023.03.13.532446; this version posted December 4, 2023. The copyright holder for this preprint (which was not certified by peer review) is the author/funder. This article is a US Government work. It is not subject to copyright under 17 USC 105 and is also made available for use under a CC0 license.

Video Legends.

Video 1. Local Spike protein injection results in neutrophil recruitment in the cremaster muscle. Confocal intravital microscopy movie shows neutrophils (green), platelets (red), endothelium (blue) immunostained with Alexa Fluor 488-Gr1, DyLight 649-GP1bβ, and Alexa Fluor 555-CD31 monoclonal antibodies, respectively. Intrascrotal injection of PBS or Spike protein (5 µg) 3-4 hours prior to imaging. The first sequence spans a ~60 min (PBS) while the second sequence spans a ~45 min (Spike) imaging period. Images were captured at ~1 frame per 30s with ~100× magnification. Time counter is hour:minute:second.

Video 2. Local injection of Spike proteins causes neutrophil fragmentation. Confocal intravital microscopy movie shows neutrophils (green), platelets (red), endothelium (blue) immunostained with Alexa Fluor 488Gr1, Dylight 649-GP1bβ, and Alexa Fluor 555-CD31 monoclonal antibodies, respectively. Intrascrotal injection of Il-1β or Il-1β plus 5 µg Spike protein ~20 hours prior to imaging. Both sequences span a 20 min period. Images were captured at ~1 frame per 30s with ~100× magnification. Time counter is hour:minute:second.

Video 3. Intravenously injected Spike protein outlines liver sinusoids and accumulates on Kupffer cells. The liver imaging was taken at three different time points; from 20 min to 1hr, from 1hr 10 min to 1hr 15 min, and from 1hr 25 min to 46 min after SARS-CoV-2 Spike protein (green, Alexa Fluor 488) injection. An image sequence of a 12 µm z-projection was acquired with 40× lens as scanning speed of 11.05 sec between frames. Kupffer cells (magenta, F4/80), and neutrophils (red, Ly6G) in liver sinusoid vasculature (cyan, CD31) were visualized with antibody injection into tail vein at 30 min prior to imaging. The scale bar represents 20 µm. Time counter is hour:minute:second.

Video 4. Neutrophils undergo NETosis when plated on A549 cells in the presence of Spike protein. A549 cells were plated on chamber slide 48 hr before trimer treatment. SARS-CoV-2 Spike trimer (green) was added to A549 cell culture 24 hr before neutrophil seeding. Purified human neutrophils stained with Hoechst (cyan) and were seeded on A549 cells in propidium iodide (PI, 1 µg/ml) containing culture media. NETosis of neutrophils were detected by exposed DNA (red, PI). An image sequence of a 20 µm zprojection was acquired with 40× lens at a scanning speed 1 frame /10 min over 5 hours. Regions of interest (Box A and B) demonstrate a typical NETosis of neutrophil contacting on trimer bearing A549 cell were enlarged in second part of video. Scale bars, 30 and 10 µm. Time banner, hour:minute:second.

bioRxiv preprint doi: https://doi.org/10.1101/2023.03.13.532446; this version posted December 4, 2023. The copyright holder for this preprint (which was not certified by peer review) is the author/funder. This article is a US Government work. It is not subject to copyright under 17 USC 105 and is also made available for use under a CC0 license.

Video 5. hSiglec-5 expressing HEK293 cells bind and endocytosis Spike protein. Individually established stable-transfected cells expressing Siglec-5-GFP or ACE2-OFP were plated in the same chamber slide with non-transfection cells. After overnight culture SARS-CoV-2 Spike protein (1 μg/ml) was overlaid for 1 hr before imaging. An image sequence of a 3 μm z-projection was acquired with 40× lens at a scanning speed of 23 sec between frames. Signals visualized as SARS-CoV-2 Spike trimer (blue), Siglec-5-GFP (green), ACE2-OFP (red), and non-transfected HEK293 cells (gray). The scale bar represents 20 μm. Time counter is hour:minute:second.

bioRxiv preprint doi: https://doi.org/10.1101/2023.03.13.532446; this version posted December 4, 2023. The copyright holder for this preprint (which was not certified by peer review) is the author/funder. This article is a US Government work. It is not subject to copyright under 17 USC 105 and is also made available for use under a CC0 license.

1070 Reagent Table

Designation	Catalog Number	SOURCE	IDENTIFIER
**Cell line**			
FreeStyle™ CHO-S Cells	R80007	ThermoFisher Scientific	
FreeStyle™ 293-F Cells	R79007	ThermoFisher Scientific	
293T	CRL-3216™	ATCC	
293 [HEK-293]	CRL-1573™	ATCC	
A549	CRM-CCL-185™	ATCC	
**Mice**			
C57BL/6J	Jax stock 000664	Jackson Lab.	IMSR_JAX:000664
K18-hACE2 (B6.Cg- Tg(K18- ACE2) 2Prlmn/J)	Jax stock 034860	Jackson Lab.	IMSR_JAX:034860
**Antibodies**			
PerCP-Cy™5.5 Rat Anti-Mouse Ly6G	560602	BD Biosciences	RRID : AB_1727563
PE-Cy™7 Rat Anti-Mouse Ly6G	560601	BD Biosciences	RRID: AB_1727562
PE Rat Anti-Mouse I-A/I-E	557000	BD Biosciences	RRID: AB_396546
PE/Cyanine7 anti-mouse CD24 Antibody	101822	BioLegend	RRID: AB_756048
PerCP/Cyanine5.5 anti-mouse CD24 Antibody	101824	BioLegend	RRID: AB_1595491
Alexa Fluor® 647 anti-mouse CD64 (FcγRI) Antibody	139322	BioLegend	RRID: AB_2566561
Alexa Fluor® 488 anti-mouse CD31 Antibody	102414	BioLegend	RRID: AB_493408

bioRxiv preprint doi: https://doi.org/10.1101/2023.03.13.532446; this version posted December 4, 2023. The copyright holder for this preprint (which was not certified by peer review) is the author/funder. This article is a US Government work. It is not subject to copyright under 17 USC 105 and is also made available for use under a CC0 license.

Alexa Fluor® 594 anti-mouse CD31 Antibody	102432	BioLegend	RRID: AB_2617017
APC-Cy™7 Rat Anti-Mouse Siglec-F	565527	BD Biosciences	RRID: AB_2732831
PE anti-mouse CD170 (Siglec-F) Antibody	552126	BD Biosciences	RRID: AB_394341
Alexa Fluor® 647 Rat Anti-Mouse Siglec-F	562680	BD Biosciences	RRID: AB_2687570
BV421 Rat Anti-Mouse Ly6C	562727	BD Biosciences	RRID: AB_2737748
Brilliant Violet 650™ anti-mouse/human CD11b Antibody	101259	BioLegend	RRID: AB_2566568
Brilliant Violet 711™ anti-mouse CD45 Antibody	103147	BioLegend	RRID: AB_2564383
Brilliant Violet 785™ anti-mouse CD11c	117336	BioLegend	RRID: AB_2565268
PE/Dazzle™ 594 anti-mouse CD19 Antibody	115554	BioLegend	RRID: AB_2564001
Brilliant Violet 421™ anti-mouse F4/80 Antibody	123137	BioLegend	RRID: AB_2563102
PE anti-mouse CD169 (Siglec-1) Antibody	142404	BioLegend	RRID:AB_109156 97
PE anti-human CD16 Antibody	360704	BioLegend	RRID: AB_2562749
PE/Cyanine7 anti-human CD56 (NCAM) Antibody	362510	BioLegend	RRID: AB_2563927
APC Mouse Anti-Human CD4	551980	BD Bioscience	RRID: AB_398521
APC/Cyanine7 anti-human CD8a Antibody	301015	BioLegend	RRID: AB_314134
Brilliant Violet 421™ anti-human HLA-DR Antibody	307635	BioLegend	RRID: AB_2561831

bioRxiv preprint doi: https://doi.org/10.1101/2023.03.13.532446; this version posted December 4, 2023. The copyright holder for this preprint (which was not certified by peer review) is the author/funder. This article is a US Government work. It is not subject to copyright under 17 USC 105 and is also made available for use under a CC0 license.

Brilliant Violet 650™ anti-human CD14 Antibody	301836	BioLegend	RRID: AB_2563799
PerCP-Cy™5.5 Mouse Anti-Human CD14	550787	BD Bioscience	RRID: AB_393884
V500 Mouse Anti-Human CD14	561391	BD Bioscience	RRID: AB_10611856
Brilliant Violet 711™ anti-human CD20 Antibody	302341	BioLegend	RRID: AB_2562602
PE/Cyanine7 anti-human CD20 Antibody	302312	BioLegend	RRID: AB_314260
Alexa Fluor® 700 anti-human CD20 Antibody	302322	BioLegend	RRID: AB_493753
PE anti-human CD123 Antibody	306006	BioLegend	RRID: AB_314580
APC/Cyanine7 anti-human CD15 (SSEA-1) Antibody	323048	BioLegend	RRID: AB_2750190
APC anti-human CD66b Antibody	305118	BioLegend	RRID: AB_2566607
Brilliant Violet 421™ anti-human CD11c Antibody	337226	BioLegend	RRID: AB_2564485
BD Pharmingen™ PerCP-Cy™5.5 Mouse Anti-Human CD3	552852	BD Biosciences	RRID: AB_394493
PE Mouse Anti-Human CD22	562859	BD Bioscience	RRID: AB_2737845
APC anti-human CD170 (Siglec-5) Antibody	352005	BioLegend	RRID: AB_2564262
APC anti-human Siglec-8 Antibody	347106	BioLegend	RRID: AB_2561402

bioRxiv preprint doi: https://doi.org/10.1101/2023.03.13.532446; this version posted December 4, 2023. The copyright holder for this preprint (which was not certified by peer review) is the author/funder. This article is a US Government work. It is not subject to copyright under 17 USC 105 and is also made available for use under a CC0 license.

Alexa Fluor® 647 anti-mouse CD169 (Siglec-1) Antibody	142408	BioLegend	RRID: AB_2563621
Human Siglec-8 Antibody	MAB7975	R & D Systems	
Human Siglec-8 PE-conjugated Antibody	FAB7975P	R & D Systems	RRID: AB_2905537
Human Siglec-1/CD169 Alexa Fluor® 647-conjugated Antibody	FAB5197R100UG	R & D Systems	RRID: AB_2905550
Human ACE-2 Alexa Fluor® 647-conjugated Antibody	FAB9332R100UG	R & D Systems	
Human Siglec-8 Alexa Fluor® 750conjugated antibody	FAB7975S-100UG	R & D Systems	
Human ACE-2 Alexa Fluor® 405-conjugated Antibody	FAB9332V-100UG	R & D Systems	
Human/Mouse/Rat/Hamster ACE-2 Antibody	AF933	R & D Systems	RRID: AB_355722
Mouse ACE-2 Antibody	AF3437	R & D Systems	RRID: AB_2223140
SARS-CoV-2 (2019-nCoV) Spike Neutralizing Antibody, Rabbit Mab	40592-R001	SinoBiological	RRID: AB_2857936
Rat IgG derivative against the GPIbβ subunit of the murine platelet/megakaryocyte-specific GPIb-V-IX complex antibody	X647	Emfret	RRID: AB_2861336
Mouse LYVE-1 Antibody	MAB2125	R & D Systems	RRID: AB_2138528
PE anti-mouse Podoplanin Antibody	127408	BioLegend	RRID: AB_2161928
**Buffer and chemicals**			
LIVE/DEAD™ Fixable Aqua Dead Cell Stain Kit	L34966	Thermo Fisher Scientific	

bioRxiv preprint doi: https://doi.org/10.1101/2023.03.13.532446; this version posted December 4, 2023. The copyright holder for this preprint (which was not certified by peer review) is the author/funder. This article is a US Government work. It is not subject to copyright under 17 USC 105 and is also made available for use under a CC0 license.

Evans Blue	E2129-10G	Sigma-Aldrich	
Propidium Iodide (PI)	P4864- 10ML	Sigma-Aldrich	
FluoSpheres™ NeutrAvidin™-Labeled Microspheres, 0.2 µm, yellow-green fluorescent (505/515), 1% solids	F8774	Thermo Fisher Scientific Inc.	
2M MgCl2	340-034-721	Quality Biological Inc.	
2M CaCl2	351-130-721	Quality Biological Inc.	
HBSS wo Ca2+&Mg2+	14175-095	Thermo Fisher Scientific Inc.	
Albumin, Bovine Serum, Fraction V, Fatty Acid-Poor, Endotoxin-Free	126579-100GM	Sigma-Aldrich	
5M NaCl	351-036-101	Quality Biological Inc.	
2M Tris-HCl, pH8.0	351-092-101	Quality Biological Inc.	
1X PBS wo Ca2+ & Mg2+	114-058-101	Quality Biological Inc.	
0.5M EDTA, pH8.0	351-027-101	Quality Biological Inc.	
Manganese (II) chloride tetrahydrate	63535-50G-F	Sigma-Aldrich	
Liberase™ TL(Thermolysin Low) Research Grade	5401020001	Roche Applied Science	
DNase I	10104159001	Roche Applied Science	
Cover glass	Cover glass – #4860-1D	Brain Research Laboratories	

bioRxiv preprint doi: https://doi.org/10.1101/2023.03.13.532446; this version posted December 4, 2023. The copyright holder for this preprint (which was not certified by peer review) is the author/funder. This article is a US Government work. It is not subject to copyright under 17 USC 105 and is also made available for use under a CC0 license.

Universal Mounting Frame AK-Set		PECON	
Opti-MEM™	31985070	Thermo Fisher Scientific Inc.	
RPMI 1640 Media	11875093	Thermo Fisher Scientific Inc.	
TransIT-293 Transfection Reagent	MIR 2704	Mirus Bio LLC	
Lenti-X™ Concentrator	631232	Takara Bio USA Inc.	
FreeStyle™ 293 Expression Medium	12338018	Thermo Fisher Scientific Inc.	
FreeStyle™ CHO Expression Medium	12651014	Thermo Fisher Scientific Inc.	
**Plasmids**			
SIGLEC5 (NM_003830) Human Tagged ORF Clone	RC206610	Origene	
SIGLEC5 (NM_003830) Human Tagged ORF Clone	RG206610	Origene	
Human Siglec-8 (NP_055257) VersaClone cDNA	RDC1496	Origene	
SARS-CoV-2 Spike-S			
HIV-1 NL4-3 Gag-iGFP ΔEnv	12455	NIH AIDS Reagent Program	
Human Coronavirus Spike glycoprotein Gene ORF cDNA clone expression plasmid(Codon Optimized) HCoV-HKU1	VG40021- UT	SinoBiological	
Human coronavirus(HCoV-229E) Spike Gene ORF cDNA clone expression plasmid(Codon Optimized) HCoV-229E	VG40605- UT	SinoBiological	
ACE2 cDNA ORF Clone, Human, C-OFPSpark® tag	HG10108-ACR	SinoBiological	

bioRxiv preprint doi: https://doi.org/10.1101/2023.03.13.532446; this version posted December 4, 2023. The copyright holder for this preprint (which was not certified by peer review) is the author/funder. This article is a US Government work. It is not subject to copyright under 17 USC 105 and is also made available for use under a CC0 license.

pCMV-dR8.2 dvpr	8455	Addgene	
pLentipuro3 TO V5-GW EGFP-Firefly Luciferase	119816	Addgene	
**Recombinant Protein**			
ACE2 Protein, Human, Recombinant (mFc Tag)	10108-H05H	SinoBiological	
Human coronavirus HKU1 (isolate N5) (HCoV-HKU1) Spike/S1 Protein (S1 Subunit, His Tag)	40602-V08H	SinoBiological	
Human coronavirus (HCoV-229E) Spike Protein (S1+S2 ECD, His Tag)	40605-V08B	SinoBiological	
SIGLEC5 Protein, Human, Recombinant (hFc Tag)	11798-H02H	SinoBiological	
Recombinant Mouse Siglec-F Fc Chimera Protein, CF	1706-SF-050	R & D Systems	
Recombinant Human Siglec-8 Fc Chimera Protein, CF	9045-SL-050	R & D Systems	

1071
